# Effect of temporal predictability on the neural processing of self-triggered auditory stimulation during vocalization

**DOI:** 10.1186/1471-2202-13-55

**Published:** 2012-05-30

**Authors:** Zhaocong Chen, Xi Chen, Peng Liu, Dongfeng Huang, Hanjun Liu

**Affiliations:** 1 Department of Rehabilitation Medicine, The First Affiliated Hospital, Sun Yat-sen University, Guangzhou, 510080, People's Republic of China

## Abstract

**Background:**

Sensory consequences of our own actions are perceived differently from the sensory stimuli that are generated externally. The present event-related potential (ERP) study examined the neural responses to self-triggered stimulation relative to externally-triggered stimulation as a function of delays between the motor act and the stimulus onset. While sustaining a vowel phonation, subjects clicked a mouse and heard pitch-shift stimuli (PSS) in voice auditory feedback at delays of either 0 ms (predictable) or 500–1000 ms (unpredictable). The motor effect resulting from the mouse click was corrected in the data analyses. For the externally-triggered condition, PSS were delivered by a computer with a delay of 500–1000 ms after the vocal onset.

**Results:**

As compared to unpredictable externally-triggered PSS, P2 responses to predictable self-triggered PSS were significantly suppressed, whereas an enhancement effect for P2 responses was observed when the timing of self-triggered PSS was unpredictable.

**Conclusions:**

These findings demonstrate the effect of the temporal predictability of stimulus delivery with respect to the motor act on the neural responses to self-triggered stimulation. Responses to self-triggered stimulation were suppressed or enhanced compared with the externally-triggered stimulation when the timing of stimulus delivery was predictable or unpredictable. Enhancement effect of unpredictable self-triggered stimulation in the present study supports the idea that sensory suppression of self-produced action may be primarily caused by an accurate prediction of stimulus timing, rather than a movement-related non-specific suppression.

## Background

A multitude of sensory stimuli are processed constantly by our sensory systems. It has been suggested that the sensory system allots more processing resources to unexpected stimuli, that often require our immediate reaction, than to stimuli that are the predicted consequences of our own actions [1]. Previous research has demonstrated that the processing of sensory consequences of self-produced actions is different from that of externally-produced stimuli [2-4]. For example, responses to self-produced tactile stimuli are suppressed relative to externally-produced stimuli in the somatosensory cortex [2,3]. The phenomenon that sensory responses to self-produced stimuli are weaker relative to externally-produced stimuli has been interpreted using an internal forward model [5,6]. The central nervous system generates an efference copy [7] of the motor command as a prediction of sensory consequences of one’s own action, and compares this prediction with the actual sensory feedback. When an accurate prediction of the actual sensory feedback is available, only a small prediction error between the intended motor action and the actual sensory feedback is generated, which in turn leads to a net cancellation of sensory input. When there is no efference copy or when the signals from the predicted and actual feedback do not match, a larger prediction error is generated, which translates to a larger response in the sensory or somatorsensory cortex.

Recently, several electrophysiological and neuromagnetic studies have demonstrated a similar suppression phenomenon in the auditory system. For example, self-triggered tones elicited suppressed event-related potentials (ERPs) or magnetoencephalography (MEG) responses as compared to the playback of the identical tones triggered by a computer [4,8-11]. Several other MEG studies in humans have also demonstrated suppressed auditory cortical responses (e.g. M100) to self-produced speech as compared to the playback of pre-recorded speech [12-17]. Ford and her colleagues conducted a series of ERP studies to investigate the efference copy mechanisms of the auditory system in normal people and patients with schizophrenia [18-20]. The results showed suppressed N1 responses to self-produced speech compared with listening to the speech playback in the normal subjects, while suppression effect was not observed in patients with schizophrenia [18,20]. In recent ERP studies where auditory feedback was pitch-shifted during vocalization, greater suppression effects (N1) of self-produced, unaltered voice were found compared with an altered voice or alien voice [21,22]. These suppression findings suggest that the auditory cortex compares the actual auditory feedback against a prediction of expected feedback to distinguish self-produced speech from externally-produced sounds.

In a literature review of auditory suppression studies, the timing of self-triggered stimulation was usually predictable. For example, pure tones were presented immediately following the actions of a participant’s button press. However, the onset of pure tones was unpredictable when they were triggered externally by the computer [9,13,17,21]. This confound leaves open the possibility that suppressed processing of self-triggered actions may be due to a fact that humans can precisely detect the temporal patterns of auditory stimuli triggered by their own actions, while the sensory consequences of externally-generated actions cannot be predicted as such. If sensory suppression did not exist when the timing of self-triggered stimulation was as unpredictable as that of externally-triggered stimulation, the suppression effect may be primarily attributed to an accurate prediction of stimulus timing. Alternatively, self-triggered stimulation could result in a general suppression of sensory events that occur with the motor act. That is, sensory suppression is due to a non-specific suppression of sensory events that relates to the motor act, and suppression of self-triggered stimulation would be independent of the delays between the motor act and the stimulus onset.

To clarify the effect of temporal predictability on the neural processing of self-trigged stimulation relative to externally-triggered one, several studies have been conducted in the auditory modality. Schafer and Marcus reported a suppression effect for N1 responses to self-triggered click sounds even when the sound onset relative to the motor act was delayed up to 4 seconds by a fixed time [8]. Bäß et al. [10] examined the cortical responses (N1) to self-triggered tones relative to the identical tones that were triggered externally, where the frequency and the onset of self-triggered tones were either predictable or unpredictable. Results showed that, even when the onset of self-triggered tones was unpredictable, N1 responses were still suppressed relative to externally-triggered tones, although the amount of suppression varied across conditions with the largest suppression for predictable frequency and predictable onset. These studies suggest that suppression of self-triggered stimulation may be due to a non-specific movement-related suppression of sensory signals.

Contrasting findings, however, were reported in several recent pitch-shifted ERP studies of self-produced vocalization [23-25]. Behroozmand et al. [23,25] reported enhanced P2 responses during active vocalization relative to passive listening when pitch-shift stimuli (PSS) were triggered by the computer with a random delay after the vocal onset (unpredictable), and only when the PSS occurred immediately after the vocal onset (predictable) did suppression effect exist [25]. Liu et al. [24] did a similar study but compared the neural responses to the self-triggered PSS with those triggered by the computer during vocalization and listening. A random delay between the mouse click and the PSS onset was introduced for the self-triggered task, and the PSS were also randomly triggered by the computer for the externally-triggered task. The results showed that unpredictable self-triggered PSS elicited larger N1/P2 responses than unpredictable externally-triggered PSS, indicating an enhancement rather than suppression effect of self-triggered stimulation [24]. These studies suggest that enhanced brain activity can be elicited to distinguish unpredictable self-triggered from unexpected externally-triggered stimulation. It should be noted, however, that several factors could possibly confound the validity of the conclusions in these studies. For instance, Behroozmand et al. [23,25] compared the neural responses during active vocalization with those during its playback; the cortical responses could be dampened due to the different physical qualities of the sounds resulting from the bone conduction during vocalization, middle ear muscle contraction, and the response characteristics of the ear [21]. One of the primary limitations in Liu et al. [24] is that the motor responses resulting from the finger movement (i. e. mouse click) were not corrected due to the lack of a motor-only task as a control condition, although the authors argued that the motor responses would not affect the neural responses to the PSS that occurred 500–1000 ms after the mouse click. This assumption, however, was not validated in all previous research. Therefore, the enhancement effect of unpredictable self-triggered stimulation observed in Liu et al.’s study [24] could be due to the effect of the motor act on the neural responses rather than the random delays between the motor act and the stimulus onset.

Given these contrasting findings, the role of temporal predictability in distinguishing self-triggered from externally-triggered stimulation is controversial. Whether sensory suppression is due to an accurate prediction of self-triggered stimulation or a direct consequence of self-triggered stimulation itself remains unclear. Therefore, the present ERP study was designed to examine the effect of temporal predictability on the neural processing of self-triggered stimulation relative to unexpected externally-triggered stimulation during self-monitoring of vocal production. The altered auditory feedback protocol [26,27] was used in this experiment: subjects vocalized a vowel sound and heard the PSS in voice auditory feedback triggered by a self- or externally-produced stimulation. Temporal predictability of self-triggered PSS was manipulated as predictable or unpredictable by introducing fixed or random delays between the motor act and the stimulus onset. For the externally-triggered stimulation, the PSS was triggered by a computer with a random delay after the vocal onset such that subjects were incapable of predicting when the PSS occurred. We expected that the temporal predictability of stimulus delivery relative to the motor act would modulate the neural processing of self-triggered stimulation relative to unpredictable externally-triggered stimulation.

## Methods

### Subjects

Seventeen right-handed, native-Mandarin speakers (8 males and 9 females) participated in this study. They reported no history of speech, hearing, neurological disorders, or voice training. All subjects passed a hearing screening at the threshold of 25 dB HL for pure-tone frequencies of 0.5-4 kHz. Four subjects were excluded from the final analysis because of excessive artifacts such as alpha activity or ocular artifacts. So data from 13 subjects (7 males and 6 females, mean age 23.3 ± 1.8 years) were finally analyzed and reported. All the subjects read and signed the informed consent approved by the Institution Review Board of The First Affiliated Hospital at Sun Yat-sen University.

### Apparatus

Subjects were seated in a sound-treated booth throughout the testing. Prior to the testing, acoustic calibration of the recording system was performed to insure that the intensity of voice feedback heard by the subjects was 10 dB (SPL) higher than that of subject’s voice output. This 10 dB gain between voice and feedback signals was used to partially mask the air-born and bone-conducted voice feedback [22,25]. Their voice signals were recorded through a dynamic microphone (Genuine Shupu, model SM-306). The microphone signal was amplified with a MOTU Ultralite Mk3 firewire audio interface and shifted by an Eventide Eclipse Harmonizer. MIDI software (Max/MSP, v.5.0 by Cycling 74), running on a Macintosh computer, was used to control the harmonizer by sending a command to produce no shift in pitch feedback or a decrease in pitch feedback to the subject. The delay time for the harmonizer to shift pitch was about 15 ms. The pitch is shifted in units of cents (cents = 100 × (39.86 × log_10_(F_0_/reference)); reference denotes an arbitrary reference note of 195.997 Hz (G4).) because this scale is logarithmically related to F_0_ and is constant relative to the absolute F_0_ of a given subject. The pitch-shifted voice signal was played back to subjects through insert plastic earphones (ER1, Etymotic Research Inc.). The voice, feedback and transistor-transistor logical (TTL) control pulses indicating the onset and offset of the stimulus were digitized at a sampling frequency of 10 k Hz by an A/D converter (PowerLab, model ML880, AD Instruments), and recorded using LabChart software (v.7.0 by AD Instruments).

### Procedures

During the experiment across all tasks, the subjects were instructed to vocalize a vowel sound (/a/, about 4 s duration) at their comfortable pitch and loudness. By instructing the subjects to vocalize without contracting speech muscles (e.g., tongue, lips or jaw), we greatly reduced the chance that such muscle contraction would affect the quality of the electroencephalogram (EEG) signals. During each vocalization, a PSS of −200 cents (100 cents = 1 semitone) with 200 ms duration was presented in voice auditory feedback. The subjects were asked to vocalize 100 times for each task and take a pause for about 3–4 seconds prior to the next vocalization to avoid vocal fatigue.

**Figure 1:**
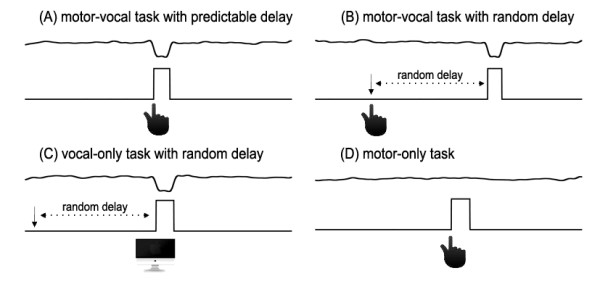
**Overview of the temporal characteristics of events in four experimental tasks.** Pitch-shift stimuli were triggered at delays of 0 ms or 500–1000 ms after mouse click for motor-vocal task with predictable delay (**A**) and motor-vocal task with unpredictable delay (**B**), respectively. Vocal-only task with random delay is illustrated in (**C**), in which a stimulus was triggered by the computer at a delay of 500–1000 ms after the vocal onset. In the motor-only task as shown in (**D**), subjects clicked the mouse but no stimulus was delivered. The traces in (**A**)-(**D**) denote pitch-shifted voice F_0_ contours (cents) and TTL control pulses indicating the onset and offset of the stimulus.

The experiment consisted of four tasks (see Figure 1). In the *motor-vocal* (MV) task, the subjects were instructed to click a mouse to trigger one PSS in the middle of the vocalization. During vocalization, they heard the PSS in voice auditory feedback at delays of 0 ms (predictable, MVP) or 500–1000 ms (random delay, MVR) after mouse click. Thus, the PSS onset was either predictable or unpredictable with respect to the motor act (mouse click). In the *vocal-only* (VO) task, the subjects vocalized the vowel while one PSS was triggered by the computer with a delay of 500–1000 ms (random delay, VOR) after the vocal onset. By doing this, the subjects were incapable of predicting the onset of the PSS. Subject’s voice onset automatically activated the MIDI program using a locally fabricated Schimitt trigger circuit that detected a positive voltage on the leading edge of the amplified vocal signals. The output of this circuit was used to trigger the PSS at a delay of 500–1000 ms with respect to the vocal onset. The timing of the MIDI output from the onset of the pulse from the vocal detection circuit was about 25 ms. In the *motor-only* (MO) task, the subjects sustained the vocalization and clicked the mouse in a similar fashion as in the MVP and MVR tasks, but no PSS were presented and their voice pitch feedback was not altered. This task was used as a control condition to rule out the motor responses resulting from the finger movement. The sequence of the intervening four tasks (MVP, MVR, VOR, MO) was randomized across all the subjects.

### EEG recording and analysis

The EEG signal was recorded with a 64-electrode Geodesic Sensor Net using a Net Amps 300 amplifier at a sampling rate of 1000 Hz (Electrical Geodesics Inc.) [28]. The electro-oculogram (EOG) artifact was monitored with four electrodes placed above and below the eyes and at the outer canthus. Individual sensors were adjusted until impedances were less than 50 kΩ [29], and all electrodes were referenced to the vertex (Cz) during recording.

After data acquisition, the EEG was re-referenced to the average of electrodes on each mastoid and band-pass filtered at 1–20 Hz. The continuous EEG was segmented into an epoch starting at 200 ms before and terminating 700 ms after the PSS onset. Segmented files were scanned for artifacts (excessive muscular activity, eye blinks and eye movements) with the Artifact Detection toolbox in Net Station (v. 4.4) using a threshold of 50 μV. Individual electrodes were rejected if they contained artifacts of any kind in more than 20% of the segments. Artifact-free segments for correct responses were averaged and baseline corrected across all conditions. The amplitudes and latencies of N1 and P2 across all tasks were measured as the negative and positive peaks in the time windows of 80–150 ms and 150–280 ms after the PSS onset.

**Figure 2:**
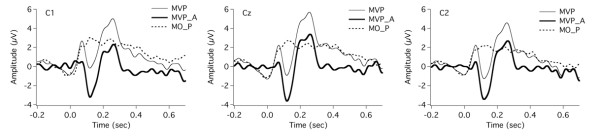
**Grand averaged ERP waveforms over all subjects at electrodes C1, Cz, and C2 for* motor-only *with zero delay (MO_P, dashed line) task, original* motor-vocal *task with zero delay (MVP, thin solid line), and corrected* motor-vocal *task with zero delay (MVP_A, thick solid line).** The response for the MVP_A condition was calculated by a subtraction of the response for the MO_P condition from that for the MVP condition.

**Figure 3:**
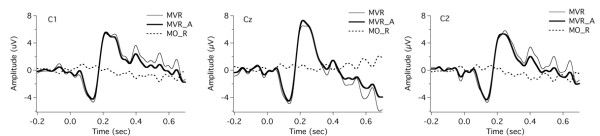
**Grand averaged ERP waveforms over all subjects at electrodes C1, Cz, and C2 for* motor-only *with random delay task (MO_R, dashed line), original* motor-vocal *task with random delay (MVR, thin solid line), and corrected* motor-vocal *task with random delay (MVR_A, thick solid line).** The response for the MVR_A condition was calculated by a subtraction of the response for the MO_R condition from that for the MVR condition.

Specifically, by using the EEG signals for the MO task, the motor responses resulting from the finger movement with respect to the PSS onset were measured to correct for the motor effect in the motor-vocal task. For the MVP task, subjects clicked the mouse and heard the PSS at a zero delay, and the TTL pulses for the MO task indicated the onset of the mouse click/PSS. Thus, averaging the segmented trials relative to the onset of the TTL pulses for the MO task led to the motor response that occurred at the onset of the mouse click, which was used to correct for the motor activity in the neural responses to the PSS for the MVP task. For the MVR task, the PSS was delivered at a random delay after the mouse click. In order to measure the motor response relative to the PSS onset, we first extracted the relative timing values indicated by the difference between the onset of mouse click and the PSS onset. These timing values were sent to the EEG trials for the MO task to generate new TTL pulses that indicated the onset of the PSS that occurred at a random delay after the mouse click. The EEG signals for the MO task were re-segmented and averaged relative to the new TTL pulses, which in turn was used to correct for the motor activity in the neural responses to the PSS for the MVR task. These two motor responses for the MVP and MVR tasks are illustrated in Figures 2 and 3 as the dashed traces labeled MO_P and MO_R. As can be seen, there is a small positive shift in the MO_P, whereas the effect of motor act on the neural response is small for the MVR task. The motor responses were subtracted from the neural responses for the MVP and the MVR task respectively, and the corrected ERPs were used for further statistical analyses.

Repeated-measures analysis of variance (RM-ANOVA) was performed on the amplitudes and latencies of N1 and P2 components using SPSS (v.16.0). Since previous research showed prominent N1/P2 responses to the PSS centrally [23,25], the amplitudes and latencies were analyzed from electrodes C1, Cz, and C2. Appropriate subsidiary RM-ANOVAs were calculated when higher-order interactions were observed. Probability values were corrected for multiple degrees of freedom using Greenhouse-Geisser if the assumption of sphericity was violated. Corrected *p* values were reported along with original degrees of freedom.

## Results

Figure 2 and 3 shows grand-averaged responses of ruling out the motor responses (MO task) from predictable and unpredictable self-triggered stimulations (MVP and MVR) at electrodes C1, Cz, and C2. The thick solid lines denote the adjusted responses (MVP_A and MVR_A), calculated by subtracting the motor responses (MO_P and MO_R) from the original responses (thin lines, MVP and MVR). In the subsequent text, the neural responses for the MVP and the MVR tasks were all corrected and tested for significance.

**Figure 4:**
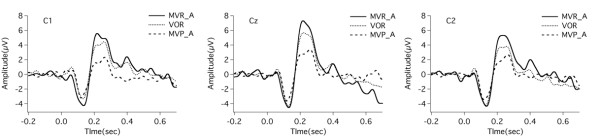
**Grand averaged ERP waveforms over all subjects at electrodes C1, Cz, C2.** The solid lines, the dotted lines, and the dashed lines denote the ERPs for the MVR, VOR and MVP tasks. The ERPs for the MVP and MVR tasks have been corrected and showed as MVP_A and MVR_A.

Figure 4 illustrates grand averaged ERP waveforms in response to the PSS across all tasks. The solid lines, the dotted lines, and the dashed lines indicate the ERP waveforms for the MVR, VOR, and MVP tasks. As shown in this figure, subjects produced similar N1 responses across all tasks, while the amplitudes of P2 responses varied as a function of task. The largest P2 amplitude was associated with the MVR task, followed by the VOR task and the MVP task.

**Figure 5:**
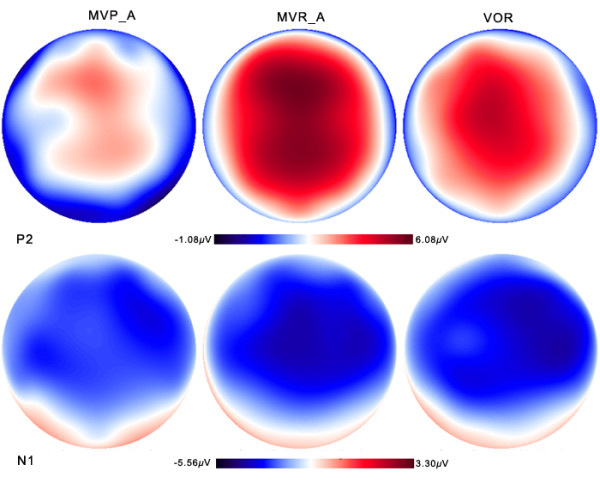
**Topographical distributions of the grand averaged ERPs to pitch feedback perturbations for the MVP (left), MVR (middle) and VOR (right) tasks.** The distributions of P2 (latency: 227 ms) and N1 (latency: 124 ms) peak amplitudes relative to the onset of pitch-shift stimulus, are shown on the top and bottom, respectively.

A task × site RM-ANOVA on N1 amplitude showed a main effect of site (F(2, 24) = 3.475, *p* = 0.047). However, the main effect of task failed to reach a significant difference (F(2, 24) = 0.102, p = 0.819). A task × site interaction was not found either (F(4, 48) = 1.521, p = 0.211). Figure 5 (bottom) shows the distributions of N1 peak amplitudes across the task illustrating the topographic differences reported above.

**Figure 6:**
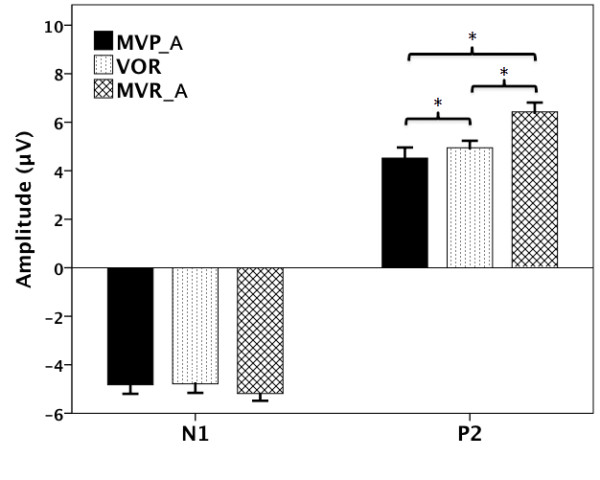
**T-bar plots showing the averaged values and standard errors of N1 and P2 amplitudes across three tasks.** The black, the dotted, and the crossed T-bars denote the responses for the MVP, VOR, and MVR task, respectively. The asterisks indicate the significant difference between conditions.

A task × site RM-ANOVA on P2 amplitude showed significant main effects of task (F(2, 24) = 17.486, *p* < 0.001) and site (F(2, 24) = 23.295, *p* < 0.001) but not for task × site interaction (F(4, 48) = 1.797, *p* = 0.145). Multiple comparisons using Bonferroni adjustment showed that the P2 amplitude in the MVR task was significantly larger than that of the MVP (p = 0.001) and VOR task (p = 0.007) (see Figure 6). In addition, the MVP task elicited significantly smaller P2 amplitudes than the VOR task (p = 0.027) (see Figure 6). Figure 5 (top) shows the P2-distribution scalp maps for the MVP (left), VOR (middle) and MVR (right) tasks, illustrating the topographic differences across three tasks.

In addition, a task × site RM-ANOVA of N1 latency (124 ± 22 ms) revealed no main effect of task (F(2, 24) = 0.860, *p* = 0.436) or site (F(2, 24) = 0.250, *p* = 0.781). Similarly, statistical results showed no systematic changes of P2 latency (227 ± 28 ms) as a function of task (F(2, 24) = 0.747, *p* = 0.485) or site (F(2, 24) = 0.756, *p* = 0.480).

## Discussion

The present study examined the modulation of the neural responses to self-triggered stimulation as a function of temporal predictability relative to externally-triggered stimulation during vocalization. The results showed that, compared with externally-triggered stimulation (VOR), P2 responses to predictable self-triggered stimulation (MVP_A) were significantly suppressed, whereas an enhancement effect was found for P2 responses to unpredictable self-triggered stimulation (MVR_A). In addition, significantly suppressed P2 responses were elicited for the MVP task as compared to the MVR task. These findings support our hypothesis that sensory consequences of self-triggered auditory events can be modulated by the temporal predictability of stimulus delivery, resulting in a suppression or enhancement effect relative to externally-triggered stimulation.

### Suppression of MVP

Sensory suppression of self-triggered stimulation has been well documented in previous research on tactile stimulation [2,3,30], speech [12-17] and hearing [4,10]. The present study is complementary to these findings by showing suppressed P2 responses to predictable self-triggered PSS compared with externally-triggered PSS during self-monitoring of vocal production. In addition, self-triggered PSS elicited suppressed P2 responses when the stimulus timing relative to the motor act was predictable than when it was not (i.e. MVP_A vs. MVR_A). Both of the results can be accounted for by the prediction-based forward model theory [6]. That is, when timing of self-triggered stimulation was fully predictable (i.e. MVP task), a precise match between the intended auditory feedback (the efference copy) and the actual auditory feedback (the re-afference) generated a cancellation of auditory inputs, leading to suppressed neural responses relative to externally-triggered stimulation. When an accurate prediction of self-triggered pitch perturbation was not available in time (i.e. MVR task), the mismatch between the intended feedback and the actual outcome generated a large prediction error, resulting in a larger brain response compared with the MVP task. Suppressed responses to predictable self-triggered PSS in the present study may reflect the allocation of neural resources away from predictable stimuli when compared with unpredictable stimuli.

### Enhancement of MVR

The major finding of the present study is that unpredictable self-triggered PSS elicited larger P2 responses than unpredictable externally-trigged PSS, leading to an enhancement effect of self-triggered stimulation. This finding reinforces one similar ERP study, showing larger P2/N1 responses to unpredictable self-triggered PSS than to unpredictable externally-triggered PSS during vocalization and listening [24]. It is also complementary to other pitch-shift studies showing the vocalization-induced enhancement effect [23,25]. Previous research in the somatosensory cortex indicated decreased sensory suppression to self-triggered tactile stimulation when the accuracy of sensory prediction in time and/or space was reduced [31]. In an analogous study, Aliu et al. [4] reported suppressed M100 responses to self-triggered tones for zero delays between the tone onset and the button press, but this suppression did not generalize to nonzero delays conditions. Results from the present study further demonstrate that, when sensory prediction to self-triggered stimulation was unavailable, neural responses were no longer suppressed but enhanced relative to unpredictable externally-triggered stimulation. This finding provides supportive evidence to the idea that sensory suppression of self-triggered stimulation may be primarily attributed to an accurate prediction of stimulus timing, rather than a movement-related nonspecific suppression of response to a sensory stimulus.

One question arising from this finding has to do with the mechanism of the enhancement effect. According to the forward model, a larger prediction error between the efferene copy and the re-afference results in a larger brain response in the sensory or somatosensory cortex [32]. For the MVR and VOR tasks in the present study, however, the forward model is unable to determine and compare the size of the prediction errors arising from these two tasks since in neither case was the stimulus delivery predictable. Thus, the enhancement effect of unpredictable self-triggered stimulation is not in line with the forward model. One plausible explanation is the selective attention related to attended and unattended times. It is suggested that that responses in the auditory cortex cannot be solely modulated by the features of acoustic stimulus but also affected by cognitive functions such as attention, memory and imagery [33]. It has been demonstrated that anticipation of an event can influence the perception of the event [34] and attention can modulate the processing of sensory inputs in both space and time [35-37]. For example, larger N1 responses were found when stimuli were presented at attended times compared with unattended times [36]. Similarly, tones presented at the attended ear elicited larger M1 responses than did unattended ear [37]. In the present study, as compared to unpredictable stimulation triggered externally, subjects may have anticipated presentation of unpredictable PSS triggered by self-initiated actions. Therefore, unpredictable self-triggered stimulation may have caused the allocation of more attentional resources, and the P2 enhancement effect may be attributed to a favored processing of auditory pitch feedback arising from more attention to self-triggered stimulation. If this speculation is true, distinguishing sensory consequences of self-triggered actions from those triggered externally may not be fully accounted for by the predicted-based internal forward model. Rather, higher cognitive function such as attention may also play an important role in this process. Supportive evidence comes from recent studies in the visual system showing that the neural responses to both predicted and unpredicted stimuli in the case of attention were larger than to the same stimuli in the absence of attention, and predicted stimuli even elicited larger responses than unpredicted stimuli in the case of attention [38]. This interaction between attention and prediction can be translated to the auditory system needs to be further explored.

### Comparison with previous studies

The enhancement effect of self-triggered stimulation in the present study is in contrast with several studies where the suppression effect was still observed when temporal predictability of self-triggered stimulation was varied [4,10]. The inconsistency between the present study and Aliu et al. [4] could be attributed to the way the temporal predictability of stimulus delivery was manipulated. In Aliu et al. [4], the tone was presented at short fixed delays (e.g. 100 ms) after button press, and subjects may not have been aware of such short fixed delays and the stimuli in these conditions were as if they occurred with a zero-delay condition (i.e. predictable). Moreover, suppression effect for nonzero delays condition was not observed when the number of trials was 100 but reached significance in the case of 300 trials, indicating a learning effect on the development of suppression of self-triggered stimulation [4]. Bäß et al. [10] reported suppressed N1 responses to self-triggered stimulation regardless of the temporal predictability of self-triggered tones. Note that their results showed significantly reduced N1 responses to unpredictable self-triggered sound onset as compared to the predictable sound onset, which was responsible for a suppression effect of the unpredictable onset of sound presentation relative to externally-triggered sound. This finding is opposite to our results and other previous studies [25,39], where neural responses to self-triggered stimulation were larger when stimulus timing was unpredictable than when it was predictable.

Another factor such as experimental task could also account for the contrasting findings between the present study and Bäß et al. [10]. The present study examined the effect of temporal predictability on the neural processing of self-triggered stimulation during self-vocalization, whereas Bäß et al. [10] addressed this effect in a listening-to-tone task. It is thus speculated that the effect of temporal predictability on the neural processing of self-triggered stimulation may be task-dependent. This speculation is supported by two recent observations. In a study examining the neural responses to PSS during active vocalization and passive listening, Behroozmand et al. [25] found that active vocalization showed enhanced P2 responses only to non-zero delayed PSS compared with passive listening, which is complementary to the enhancement effect of unpredictable self-triggered stimulation in the present study. By contrast, Lange [40] compared the N1 response to self-triggered tones with those to equally predictable visually cued tones and found suppressed N1 responses to self-triggered tones irrespective of whether the onset of sound presentation was predictable or not, which parallels the results reported by Bäß et al. [10]. Therefore, there may be a task-dependent manner of temporal predictability on the neural processing of self-triggered stimulation. Whether the present findings can be translated to the listening-related tasks remains to be investigated.

In addition, the PSS across three vocal tasks were presented to voice pitch feedback at different times with respect to the onset of vocalization, which could confound the results. In previous research of the efference copy mechanisms during speech [13,21-23], the acoustic stimuli occurred at the same time relative to the vocal onset during active vocalization and passive listening. In the present study, however, the variability in the PSS presentation relative to the vocal onset was unavoidable because of the difference between self-paced mouse click and the computer triggering. In order to rule out this confounding factor, future experiments should be conducted to testify the effect of the delay between the vocal onset and stimulus presentation on the neural processing of voice pitch feedback during vocalization.

### N1 vs. P2

In the present study, the suppression/enhancement effect of self-triggered stimulation was reflected in the P2 response. Similarly, other pitch-shift ERP studies also showed the modulation of neural responses to pitch perturbation at P2 across tasks [23,25,41]. By contrast, previous auditory research shows prominent suppression effect at N1 [8,10,11,39,40]. It is speculated that the observation of a P2 effect in the present study and a N1 effect in other studies can be due to the specific demands of different tasks. In the present study, subjects sustained a vowel phonation and heard their pitch feedback shifted at the mid-utterance. It has been demonstrated that subjects compensate for pitch errors by changing their voice pitch in the opposite direction to the stimuli [26,42]. It has been suggested that P2 has multiple generators with a center of activity near Heschl’s gyrus [43], which overlaps the neural network for the correction of pitch errors during vocal motor control [44,45]. Thus, given the involvement of those sources of P2 generation in the vocal motor control, it may account for the P2 effect observed in the present study as well as other pitch-shift studies during vocalization [23,25,41].

It is also noted that some pitch-shift studies reported N1 suppression of active vocalization relative to passive listening [21,22], but this suppression only occurred when the PSS was presented at the vocal onset. If it was presented in mid-utterance, an enhancement effect was found for P2 only [25]. This could be due to the differential mechanisms of vocal motor control at utterance onset and mid-utterance [46]. At utterance onset, auditory feedback is compared with the efference copy predicted by a forward model to reach a pre-planned pitch goal, and a prediction error results in a suppression effect that is similar to other listen-to-tone studies. It has been suggested that N1 has multiple generators in primary and secondary auditory cortex [47,48] and is considered to reflect the automatic detection of acoustic changes [49], which may account for the prominent suppression effect at N1. Therefore, the lack of an N1 effect in the present study does not challenge previous findings regarding N1 suppression of self-triggered tones, but our speculation of task dependency in the suppression/enhancement of the N1/P2 during self-triggered stimulation needs to be further explored.

## Conclusion

The present study examined the differential processing of self- and externally-triggered stimulation as the delays between the motor act and the PSS onset were manipulated during self-monitoring of vocal production. As compared to unpredictable externally-triggered PSS, P2 responses to predictable self-triggered PSS were suppressed, whereas an enhancement effect for P2 responses was found in the case of unpredictable self-triggered PSS. These results demonstrate that the neural processing of self-triggered stimulation can be modulated as a function of temporal predictability of stimulus onset related to the motor act. The finding of an enhancement effect for unpredictable self-triggered stimulation provides supportive evidence to the idea that suppression of self-triggered stimulation could be primarily due to an accurate prediction of stimulus timing rather than a movement-related nonspecific suppression.

## Authors’ contributions

ZC and HL conceived and designed the experiments. ZC, XC, and PL performed the experiment and analyzed the data. ZC and DH contributed the materials and analysis tools. ZC, DH, and HL wrote the paper. All the authors read and approved the final draft.
